# Peptide signaling at the intersection of growth, nutrient sensing, and stress responses

**DOI:** 10.1111/tpj.70733

**Published:** 2026-02-15

**Authors:** Patricia Zecua‐Ramirez, Vikram Jha, Martin Stegmann

**Affiliations:** ^1^ Molecular Botany, Institute of Botany Ulm University 89081 Ulm Germany

**Keywords:** peptide signaling, phytocytokines, receptor kinases, signal integration

## Abstract

Plant endogenous peptides emerge as central regulators of multiple aspects of plant physiology. They are secreted from cells and perceived by plasma membrane localized receptors, which activate downstream signaling pathways to regulate growth and development. In addition, endogenous peptides coordinate physiology with their environment. This includes nutrient sensing and stress responses, ranging from adaptations to adverse abiotic environments to the regulation of immune responses. Members of several endogenous peptide families exhibit versatile physiological functions, raising the question of whether they coordinate multiple endogenous and exogenous signals to confer physiological plasticity. In this review, we discuss recent insights into the multifacetted roles of peptides at the intersection of growth, nutrient sensing, and stress responses.

## INTRODUCTION

Plants tightly control their growth and development to optimize physiological needs to their sessile lifestyle. In nature, environmental conditions are dynamic with fluctuations in water and nutrient availability, as well as daily and seasonal variations in light intensity and temperature. As a consequence, plant growth and development is strongly influenced by abiotic conditions. Plants are colonized above and below ground by numerous microbes, including commensals and mutualists that collectively form the microbiome, with tremendous implications for plant fitness (Bhat & Haney, 2025). Furthermore, plants are constantly challenged by potential pathogens that attempt to infect host tissue to extract nutrients.

To monitor their surroundings, plants evolved plasma membrane receptors, often receptor kinases (RKs) with intracellular kinase domains that recognize non‐self‐molecules and activate appropriate downstream outputs. Plants employ pattern recognition receptors (PRRs) to detect potential invaders. PRRs recognize microbe‐associated molecular patterns (MAMPs) to mount pattern‐triggered immunity (PTI), a basal resistance response to fend off non‐adapted pathogens (DeFalco & Zipfel, 2021; Ngou et al., 2022). Symbiotic organisms, including root nodule bacteria and arbuscular mycorrhizal fungi (AMF), secrete lipochitooligosaccharides that are perceived by plasma membrane RKs to initiate symbiosis programs (Chiu & Paszkowski, 2020). To increase complexity, symbiont, and pathogen recognition share similar receptors and mechanisms that determine specificity and are only beginning to be unraveled (Tsitsikli et al., 2025).

PRR complexes recruit co‐receptors to initiate downstream signaling, including phosphorylation cascades, cytosolic influx of calcium ions, production of reactive oxygen species, and transcriptional reprogramming (DeFalco & Zipfel, 2021). Local immune activation is translated into systemic responses, involving biosynthesis and signaling of salicylic acid (SA), jasmonic acid (JA), and ethylene (Vlot et al., 2021).

Adapted pathogens can overcome PTI by secreting effectors that block PTI activation or interfere with signal transduction. Such perturbations in turn can be perceived by intracellular NUCLEOTIDE‐BINDING LEUCINE‐RICH REPEAT (NLR) proteins to activate effector‐triggered immunity (ETI) (Ngou et al., 2022). NLRs form multimeric resistosome complexes that converge on cell death execution to counteract colonization by biotrophic pathogens (Ngou et al., 2022).

Excessive immune activation strongly affects plant growth and development, similar to adverse abiotic conditions. This is known as the growth–defense trade‐off (Hou & Xu, 2025). This necessitates the existence of mechanisms that integrate multiple external and internal cues to ensure physiological plasticity. Understanding these mechanisms and their molecular wiring may help engineer plants for more sustainable agriculture.

Plant endogenous signaling peptides emerge as central regulators of most (if not all) aspects of plant physiology and act in concert with classical phytohormones by mutual cross‐regulation. In contrast to phytohormones, plant endogenous peptides are encoded by single genes and translated into polypeptide chains that are mostly targeted to the secretory pathway for apoplast delivery (Lalun & Butenko, 2025). Their biosynthesis, however, is similarly complex, involving sometimes multiple steps of post‐translational modifications (PTMs). This includes proteolytic release of the mature peptide from its precursor by endogenous proteases, often involving SUBTILISIN‐LIKE SERINE PROTEASES (SBTs) and frequently additional post‐translational modifications, such as proline hydroxylation, tyrosine sulfation, or hydroxyproline arabinosylation (Lalun & Butenko, 2025).

**Figure 1:**
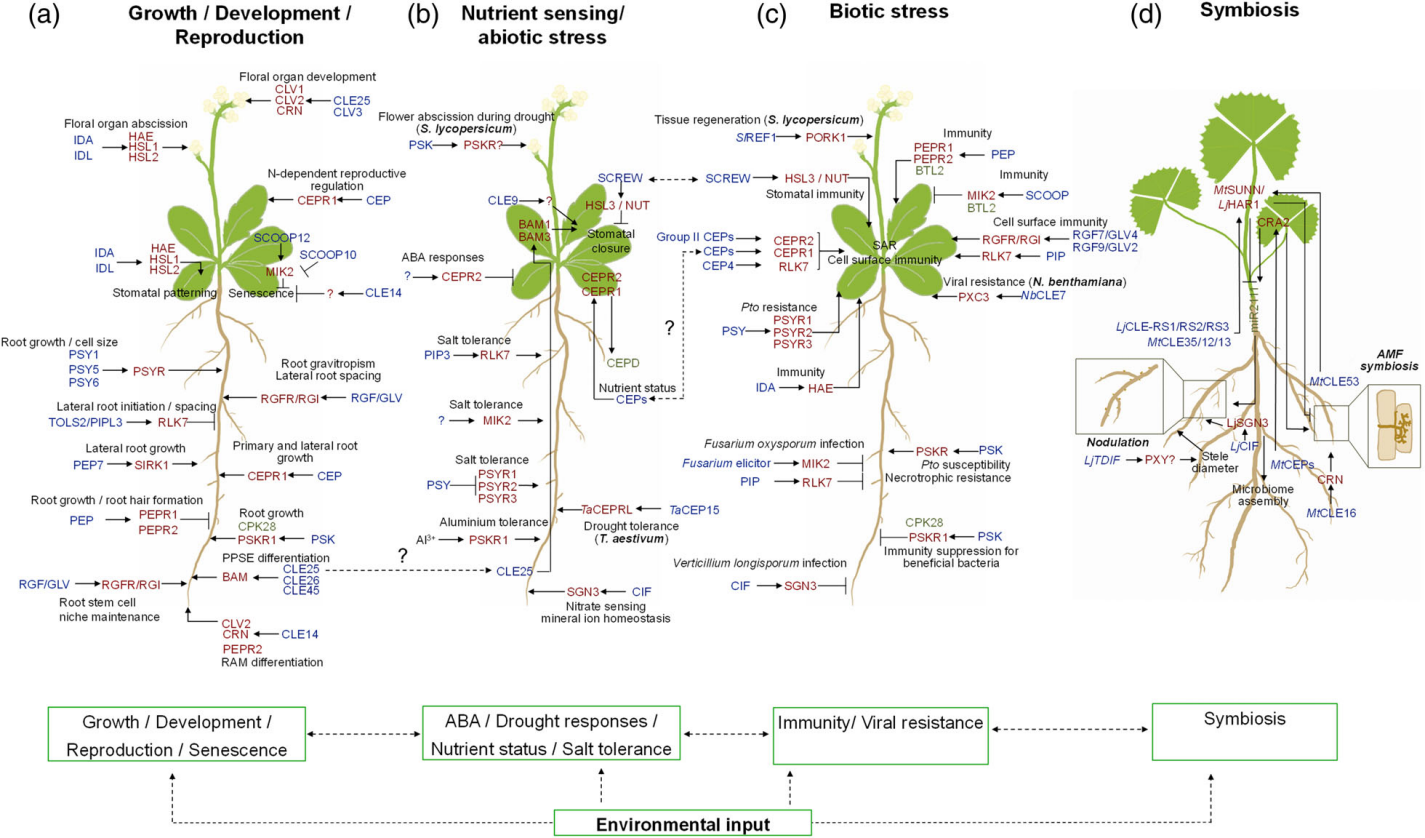
Endogenous signaling peptides at the interface of plant growth and stress responses. Endogenous peptides operate at the intersection of growth/developmental programs, nutrient sensing, stress responses and plant–microbe interactions. (a) Peptide pathways associated with growth, development and reproduction. (b) Peptide modules implicated in abiotic stress responses and nutrient homeostasis. (c) Peptide modules associated with plant immunity and biotic stress. (d) Peptide pathways associated with symbiosis in legume species. (a–c) If not indicated otherwise, peptide receptor modules were described in Arabidopsis. Many peptide families act across multiple contexts and may integrate diverse endogenous and environmental cues. Peptide families or representative members are depicted in blue; known perceiving receptors in red and additional key signaling components in green. Solid arrows denote positive regulation, headless arrows negative regulation. Dashed connections indicate potential crosstalk across pathways.

Upon arrival in the apoplast, peptides bind to plasma membrane RKs that are structurally similar to PRRs and often belong to the leucine‐rich repeat receptor kinase (LRR‐RK) family (Hohmann et al., 2017). Akin to MAMP‐PRR pathways, peptide‐RK modules form receptor‐co‐receptor complexes and induce downstream responses that rely on the recruitment of very similar or even identical proteins (DeFalco et al., 2022; Hohmann et al., 2017). Peptides that modulate immunity are referred to as phytocytokines, in analogy to metazoan cytokines (Rzemieniewski & Stegmann, 2022). In this review, we summarize emerging roles of peptides at the intersection of growth, nutrient sensing, stress responses, and plant–microbe interactions (Figure 1) and discuss important directions for future research (Box 1).

Box 1 **Bullet‐point summary**

- An increasing amount of peptide modules previously associated with regulation of growth and nutrient homeostasis emerge as coordinators of biotic and abiotic stress responses.
- Many peptide receptor modules converge on similar molecular executors to affect diverse physiological outputs.
- Novel functions of peptide modules emerge, including tissue regeneration and dual functionalities as structural and signaling components, as well as novel roles for receptor kinase domains in ligand perception.
- Peptide perception shows enormous diversity: individual receptors perceive multiple (agonist and antagonist) ligands, while other receptors cooperate to recognize distinct peptide cocktails.
- Accumulating examples demonstrate conservation of peptide signaling pathways across species with potential crop improvement applications.

## CLE PEPTIDES AS REGULATORS OF MERISTEM ACTIVITY, SYMBIOSIS, AND DROUGHT STRESS RESPONSES

CLAVATA 3/EMBRYO SURROUNDING REGION (CLE) peptides have central roles in the regulation of meristematic activity. Generally, CLEs bind to LRR‐RKs of the CLAVATA1 (CLV1)/BARELY ANY MERISTEM (BAM) family or the RECEPTOR‐LIKE PROTEIN (RLP) CLAVATA2 (CLV2) in complex with the pseudokinase CORYNE (CRN). Upon CLE binding, receptors recruit CLV‐INSENSITIVE RECEPTOR KINASE (CIKs) to control stem cell differentiation in root/shoot meristems and vascular development (Eswaran et al., 2024; Selby & Jones, 2023). CLEs also control biotic and abiotic stress tolerance. In *Arabidopsis thaliana* (hereafter Arabidopsis), CLE25 is produced in roots upon drought stress and travels to the shoot where perception by BAM1 and BAM3 promotes expression of the abscisic acid (ABA) biosynthesis enzyme NCED3 to promote ABA accumulation and stomatal closure (Takahashi et al., 2018). CLE25 also suppresses protophloem sieve element (PPSE) differentiation and regulates floral organ development (Hu et al., 2021; John et al., 2023; Jones et al., 2021; Qian et al., 2022). It remains unknown how developmental roles are integrated with drought stress responses and/or ABA signaling. It is conceivable that CLEs are employed to adapt development and/or root growth based on environmental inputs (Fukuda & Hardtke, 2020). *CLE25* and the related *CLE26* show overlapping expression patterns with *CLE45* in the root and redundantly regulate PPSE differentiation (Gujas et al., 2020; Qian et al., 2022). In contrast to CLE26, the effect of CLE45 signaling through BAM3 is compromised at alkaline pH, which may represent a regulatory circuit to buffer CLE sensitivity across pH modulated PPSE developmental trajectories (Diaz‐Ardila et al., 2023). CLE9 is expressed in stomata and also regulates drought stress tolerance via ABA signaling (Zhang et al., 2019). Whether and how paracrine CLE25 is integrated with autocrine CLE9 for optimal outputs remains unknown.

CLE14 inhibits chlorophyll degradation and senescence by promoting ROS detoxification (Zhang et al., 2022). CLE14 also controls nutrient status‐dependent growth responses. CLE14 is perceived by CLV2‐CRN and PEP RECEPTOR 2 (PEPR2) to promote root apical meristem differentiation upon phosphate starvation resulting in root growth arrest (Gutiérrez‐Alanís et al., 2017). Whether these receptor(s) are also involved in CLE14 perception during senescence remains unknown (Gutiérrez‐Alanís et al., 2017; Zhang et al., 2022).

Emerging evidence connects CLE signaling to the regulation of plant–microbe interactions, including symbiosis and plant immune responses against pathogens. CLE peptides regulate root nodule and AMF symbiosis in legumes and other plant species (Ascurra & Müller, 2025). For example, *Medicago truncatula* CLE12, CLE13, and CLE35 suppress nodulation in a root‐to‐shoot communication pathway to balance nodulation with nutrient availability (called autoregulation of nodulation). Root‐derived CLE35 is perceived by the shoot‐expressed CLV1 homolog SUPER NUMERIC NODULES (SUNN), a negative regulator of nodulation (Gautrat et al., 2020; Laffont et al., 2019; Mens et al., 2021). In *Lotus japonicus*, a similar function is carried out by the orthologous HYPERNODULATION ABERRANT ROOT FORMATION (HAR1) that perceives CLEs, including CLE‐RS1, CLE‐RS2, and CLE‐RS3 to suppress nodulation (Krusell et al., 2002; Nishimura et al., 2002; Okamoto et al., 2013). Interestingly, *CLE35* expression is induced by both nitrate and Rhizobia, while *CLE12*/*CLE13* abundance is not sensitive to nitrate supplementation (Mens et al., 2021). This raises the question of whether distinct CLE peptides can modulate symbiosis independent of nutrient status, possibly as part of an immune response to avoid over colonization.

Plants employ similar mechanisms to balance AMF colonization. For example, root vasculature expression of *Medicago truncatula CLE53* is responsive to AMF and suppresses colonization via SUNN (Müller et al., 2019). By contrast, the cortex‐expressed CLE16 promotes AMF symbiosis via CRN in the same species (Bashyal et al., 2025). TRACHEARY ELEMENT DIFFERENTIATION FACTORs (TDIFs) are specific CLEs associated with vascular patterning (He et al., 2025). *Lotus japonicus* TDIF enhances stele diameter and promotes nodulation, suggesting an interplay between vascular development and symbiosis. Collectively, multiple CLEs emerge as positive and negative regulators of symbiosis, but their molecular interplay remains largely unknown (de la Teyssendier Serve et al., 2025).

A direct role of CLEs for plant immunity was recently revealed for the *Nicotiana benthamiana* CLE7, which is secreted upon infection with the viral pathogen Chinese wheat mosaic virus (CWMV) and perceived by PXC3, a BAM1/CLV1 homolog, to induce antiviral immunity (Liu, Zhang, et al., 2024).

CLEs emerge as central regulators of diverse stress responses, integrating nutrient and water status with (symbiotic) plant–microbe interactions and (antiviral) immunity. Given the large amount of CLE peptides encoded in plant genomes, for example, 33 in Arabidopsis and 52 in tomato, it is conceivable that additional roles in stress responses emerge (Selby & Jones, 2023). Yet, how the stress‐related functions are coordinated with their canonical developmental roles requires further investigations but raises interesting implications for plant morphological adaptions to optimize physiology to diverse environmental inputs.

## TYROSINE‐SULFATED PEPTIDES AT THE NEXUS OF THE GROWTH‐STRESS TRADE‐OFF

Tyrosine sulfation is an essential post‐translational modification in several plant‐secreted peptides and is catalyzed by TYROSYL PROTEIN SULFOTRANSFERASE (TPST) (Kaufmann & Sauter, 2019). PHYTOSULFOKINEs (PSKs) are TPST substrates that regulate multiple growth‐related processes, including cell differentiation, root elongation, and pollen tube germination/elongation (Kaufmann & Sauter, 2019). PSK maturation further requires SBT‐mediated precursor cleavage (Reichardt et al., 2020). Upon secretion, PSKs bind to the LRR‐RKs PSK RECEPTOR 1 (PSKR1) and PSKR2 in a sulfotyrosine‐dependent manner to recruit SOMATIC EMBRYOGENESIS RECEPTOR KINASE (SERK) co‐receptors, including BRI1‐ASSOCIATED KINASE 1 (BAK1) (Wang et al., 2015). PSK signaling is an important regulator of plant immunity by differentially affecting disease resistance against microbes with distinct lifestyles. PSK represses PTI and resistance against the hemibiotrophic bacterial pathogen *Pseudomonas syringae pv. tomato* (*Pto*), but promotes resistance against the necrotrophic fungi *Botrytis cinerea* and *Alternaria brassicicola* in Arabidopsis and tomato (Ding, Lv, et al., 2022; Igarashi et al., 2012; Mosher et al., 2013; Zhang et al., 2018).

PSK signaling recently emerged as a key node regulating the growth–defense trade‐off. PSKR1 shapes the microbiome by promoting root colonization of beneficial *Pseudomonas fluorescens* through suppression of SA‐dependent immunity (Song et al., 2023). PSKR1 interacts with CALCIUM‐DEPENDENT PROTEIN KINASE 28 (CPK28), which is required for PSK‐dependent growth promotion and immune suppression (Ding, Lv, et al., 2022). The PSKR1‐CPK28 module promotes phosphorylation of the key nitrogen assimilation enzyme GLUTAMINE SYNTHASE 2 (GS2). CPK28 phosphorylates GS2 at two distinct sites, Serine 334 and Serine 360. S334 phosphorylation is important for PSK‐induced immune suppression, while pS360 is important for root growth promotion. Interestingly, a GS2^S334D^ mutant mimicking S334 phosphorylation retains PSK‐induced growth, shows constitutively increased resistance against *Pto*, and is insensitive to the PSK resistance inhibitory effect. However, it remains to be tested whether this mutant may increase susceptibility against pathogens with different lifestyles. CPK28 is a negative regulator of PTI and buffers the levels of the central immune regulatory RECEPTOR‐LIKE CYTOPLASMIC KINASE (RLCK) BOTRYTIS‐INDUCED KINASE 1 (BIK1) (Monaghan et al., 2014). CPK28 further promotes nitrate uptake under nitrogen deprivation via phosphorylation of the NITRATE TRANSPORTER 2.1 (NRT2.1), as well as phosphorylation and nuclear translocation of the transcription factor and nitrate receptor NIN‐LIKE PROTEIN 7 (NLP7) during cold stress (Ding, Yang, et al., 2022; Liu, Liu, et al., 2022; Yu et al., 2025). This highlights the central role of CPK28 for the adaptation to stress responses and their integration with plant growth. An exciting avenue for future research is to understand which of these multifunctional CPK28 roles are attributed to the PSK‐PSKR1 pathway.

PSK signaling is involved in abiotic stress responses, including drought stress in tomato and Arabidopsis (Reichardt et al., 2020; Stührwohldt et al., 2021). PSKR1 also senses toxic aluminum ions and triggers, among other components, a PP2C‐TYPE PHOSPHATASE H1 (PP2CH1)/PP2CH2‐dependent signaling pathway converging on organic ion secretion for extracellular aluminum sequestration (Cao et al., 2025; Ding et al., 2024; Ma et al., 2025; Xu et al., 2025). Interestingly, PSKR1 recognizes aluminum via its intracellular kinase domain and an interplay with canonical extracellular PSK perception remains unknown. However, *pp2ch1/2* mutants display loss of PSK‐induced root growth promotion, suggesting mechanistic interdependence. Collectively, PSKs are important regulators of plant growth and diverse stress responses by sensing extracellular peptides and accumulation of toxic intracellular ions. Understanding the underlying mechanisms could tip the balance toward more efficient growth without compromising stress tolerance in plants.

PLANT PEPTIDE CONTAINING SULFATED TYROSINEs (PSYs) are 14–21 amino acids in length and substrates for TPST to induce sulfation of a conserved tyrosine residue (Kaufmann & Sauter, 2019; Ogawa‐Ohnishi et al., 2022). PSY1 induces several growth‐related outputs, including an increase in cell size and root growth (Kaufmann & Sauter, 2019). Recently, eight additional related peptides (designated as PSY2‐PSY9) were identified in Arabidopsis (Ogawa‐Ohnishi et al., 2022). The LRR‐RK subfamily X member PSY1R has initially been proposed as a PSY1 receptor by partial loss of PSY1 sensitivity in *psy1r* mutants (Amano et al., 2007). However, recently, three LRR‐RK subfamily XI emerged as bona fide PSY receptors. Photoactivatable peptide cross‐linking revealed PSY binding to PSYR1, PSYR2, and PSYR3, while PSY1R did not display direct PSY interaction (Ogawa‐Ohnishi et al., 2022). PSY5 application and PSY6 overexpression enhanced root growth in Arabidopsis, suggesting similar growth regulatory roles. Interestingly though, a *psyr1/2/3* triple mutant displayed enhanced root growth, despite its function as a receptor for growth promoting peptides. Instead, the *psyr1/2/3* mutants displayed increased sensitivity to salt treatment and enhanced susceptibility to *Pto* infection. Supported by transcriptomic data, the authors proposed a model in which constitutively expressed PSY peptides repress PSYR1‐3 function to allow growth, while stress‐associated tissue damage results in local PSY depletion to de‐repress PSYRs. *PSY1R* mutants display enhanced resistance to *Pto* (Mosher et al., 2013), raising the question of how PSY1R and PSYR1‐3 interact to regulate stress and growth.

ROOT MERISTEM GROWTH FACTOR (RGFs), also known as GOLVEN (GLV) or CLE‐LIKE (CLEL) peptides, are perceived by RGF RECEPTORs/RGF INSENSITIVEs (RGFR/RGI) to regulate root stem cell niche maintenance via ROS‐regulated abundance control of the transcription factor PLETHORA (Lu et al., 2020; Shao et al., 2020; Yamada et al., 2019). RGI‐RGF‐MITOGEN ACTIVATED PROTEIN KINASE (MAPK) signaling further regulates root gravitropism and lateral root spacing (Fernandez et al., 2020). Recent insights reveal that RGF/GLV/CLELs are also implicated in plant immunity, but it remains unknown how this is coordinated with their canonical growth regulatory functions. A particular member, RGF7/GLV4, is transcriptionally induced upon pathogen infection and treatment with the immunogenic elicitor flagellin 22 (flg22). Inducible RGF7 overexpression induced MAPK activation and defense gene expression in an RGI‐dependent manner, but application of predicted synthetic peptides was ineffective, leaving the question of the true identity of the bioactive peptide (Wang et al., 2021). In addition, further RGF peptides were shown to control immunity. RGF9/GLV2‐RGI signaling promotes the abundance of the flg22 receptor FLAGELLIN SENSING 2 (FLS2), and higher order *rgi* mutants are more susceptible to *Pto* infection (Stegmann et al., 2022; Wang et al., 2021).

CASPARIAN STRIP INTEGRITY FACTORs (CIFs) are critical regulators of barrier integrity, including formation of the casparian strip. CIFs have been identified as root stele‐expressed substrates of TPST. CIFs are perceived by the LRR‐RK SCHENGEN 3 (SGN3) which induces activation of the RLCK SGN1 to induce CASPARIAN STRIP DOMAIN PROTEIN (CASP) fusion and barrier closure (Doblas et al., 2017; Nakayama et al., 2017). Accumulating evidence suggests a role for CIF‐SGN signaling for abiotic stress tolerance and plant–microbe interactions. SGN pathway mutants show defects in nitrate sensing, mineral ion homeostasis and defective microbiome assemblies by mis‐regulated glutamine secretion (Nakayama et al., 2017; Salas‐González et al., 2021; Shen et al., 2024; Tsai et al., 2025). In *Lotus japonicus*, SGN signaling links root‐to‐shoot communication for effective nodulation (Shen, Micic, et al., 2025). CIF signaling is also involved in restricting vascular infection by the fungal pathogen *Verticillium longisporum* (Fröschel et al., 2021), suggesting that CIFs emerge as hubs connecting nutrient status, microbiome homeostasis and immunity.

## SCOOP PEPTIDES CONNECTING IMMUNITY WITH CELL WALL INTEGRITY SENSING

SERINE‐RICH ENDOGENOUS PEPTIDES (SOOPs) are produced upon stress and trigger typical PTI‐like signaling outputs (Hou et al., 2021; Rhodes et al., 2021; Yang et al., 2023). SCOOPs are specific to Brassicales with 50 members in Arabidopsis (Yang et al., 2023). The active epitope of SCOOPs is generally predicted to be 13 amino acids in length with a conserved SxS motif (Hou et al., 2021; Rhodes et al., 2021). SCOOPs contain SBT cleavage sites and several SBTs could cleave PROSCOOP precursors (Yang et al., 2023). Interestingly, despite the large number of SCOOP precursor genes in Arabidopsis, their perception relies on a single LRR‐RK, MALE DISCOVERER 1‐INTERACTING RECEPTOR‐LIKE KINASE 2 (MIK2) in complex with BAK1 (Hou et al., 2021; Rhodes et al., 2021). The mechanism of SCOOP perception by MIK2‐BAK1 has been predicted by artificial intelligence‐based structural modeling with comparative genomics, and recently confirmed by crystallization, showing that the conserved SxS motif is essential for SCOOP‐MIK2 binding (Snoeck et al., 2024;Sun et al., 2024; Wu et al., 2024). The crystal structure revealed a glycosylated asparagine residue on MIK2 that is essential for complex formation with BAK1 upon ligand binding, revealing a previously undescribed recognition mechanism for endogenous peptides (Sun et al., 2024; Wu et al., 2024). MIK2 is an important component of the plant immune system, illustrated by increased susceptibility of *mik2* mutants to the fungal pathogen *Fusarium oxysporum* (*Fox*) and the herbivory insect *Spodoptera littoralis* (Stahl et al., 2022; Van der Does et al., 2017). In addition, accumulating evidence suggests that MIK2 is important for development, including senescence and root skewing with those roles linked to cell wall integrity signaling (Van der Does et al., 2017; Zhai et al., 2024; Zhang et al., 2024). Inhibition of cellulose biosynthesis by the herbicide isoxaben (ISX) induces cell wall damage and ISX‐induced transcriptomic changes mimic PTI to a large extent (Zhai et al., 2024). ISX promotes *SCOOP18* expression to increase abundance of several PTI signaling components (DeFalco & Zipfel, 2021; Zhai et al., 2024). The *mik2* mutant shows increased senescence. SCOOP12 perception by MIK2 delays, while SCOOP10 accelerates senescence in a MIK2‐dependent manner (Zhang et al., 2024). Both SCOOPs compete for binding to the extracellular domain of MIK2. SCOOP12 promotes, while SCOOP10 reduces MIK2 phosphorylation and activity. This suggests that at least two SCOOP peptides display receptor antagonistic functions (Zhang et al., 2024). Given the large number of SCOOPs, it is conceivable that other physiological outputs are fine‐tuned by antagonistic SCOOPs. It will be important to reveal how SCOOPs can coordinate stress responses with development, potentially by determining precise signaling outputs through spatiotemporal availability of antagonistic ligands (Steidele et al., 2025).

Interestingly, the majority, but not all, MIK2 functions can be attributed to SCOOP perception. The large number of SCOOP genes in Arabidopsis with 50 members hamper genetic characterization of individual SCOOP's physiological roles. However, an octuple mutant that eliminates all potential SCOOP maturating SBTs phenocopied *mik2* in most physiological contexts, for example, ISX‐induced stress gene expression and enhanced *S. littoralis* susceptibility (Yang et al., 2023). However, the enhanced susceptibility of *mik2* to *Fox* appears independent from endogenous SCOOPs (Yang et al., 2023). MIK2 also perceives an yet undescribed elicitor derived from *Fox* extracts, suggesting that a single receptor perceives both exogenous and endogenous patterns to regulate plant immunity (Coleman et al., 2020). *Fox* also contains predicted SCOOP sequences, but whether these are causative for bioactivity of extracts remains to be determined (Hou et al., 2021). Interestingly, MIK2 orthologs can be identified in various non‐Brassicales plant species, including tomato. In tomato, MIK2 can perceive the *Fox* elicitor, but does not respond to Arabidopsis SCOOP peptides, suggesting the ability to detect distinct exogenous and endogenous patterns (Maroschek et al., 2025). It will be important to combine biochemistry with genetics to determine the exact nature of the *Fox*‐derived elicitor perceived by MIK2 to better decipher its dual function for self and non‐self‐recognition.

## CEP PEPTIDES AS COORDINATORS OF GROWTH, SYMBIOSIS, IMMUNITY, AND ABIOTIC STRESS RESPONSES

C‐TERMINALLY ENCODED PEPTIDEs (CEPs) are derived from larger precursors that can carry multiple CEP domains (Delay et al., 2013). *CEPs*' expression is sensitive to several environmental factors, including low nitrogen, high carbon, and salt (Delay et al., 2013). CEPs integrate carbon and nitrogen status to regulate primary and lateral root elongation and root system architecture (Taleski, Jin, et al., 2023; Taylor et al., 2025).

In roots, CEPs are induced upon nitrogen (N) starvation, translocated to the xylem and transported to the shoot. Here, they bind to two closely related LRR‐RKs, CEP RECEPTOR 1 (CEPR1), and CEPR2, resulting in the production of shoot‐to‐root mobile CEP DOWNSTREAMs (CEPDs) glutaredoxins (GRXs) (Ota et al., 2020; Tabata et al., 2014). CEPDs are transported to the root where they modulate nitrogen transporter *NRT2.1* expression and CEPD‐INDUCED PHOSPHATASE (CEPH)‐dependent NRT2.1 dephosphorylation, thus transcriptionally and post‐translationally modifying NRT2.1‐dependent nitrate uptake (Ohkubo et al., 2021; Tabata et al., 2014). CEPDs regulate the activity of the TGACG (TGA) transcription factors TGA1/4. In the absence of CEPDs, TGA1/4 form a transcriptional repressor complex with ALWL motif‐containing GRXs and TOPLESS. CEPDs outcompete ALWL‐GRXs to induce transcription of N starvation genes, including *NRT2.1* (Kobayashi et al., 2024; Thurow et al., 2025). CEP signaling depends on cytokinin responses which both converge on CEPDs to regulate root growth (Taleski, Chapman, et al., 2023). The role of CEPR2 in these processes is not clear, as illustrated by strong phenotypes in *cepr1* mutants that are not markedly promoted upon additional loss of *CEPR2* (Tabata et al., 2014; Taleski, Jin, et al., 2023). However, shoot‐expressed CEPR2 was recently demonstrated to perceive root‐derived CEP1 to induce ROS‐dependent stomatal closure upon N starvation. This suggests a role for CEPR2 in coordinating shoot CO_2_ uptake for photosynthesis with root N availability (Wang, Chen, Shi, et al., 2025). CEPR1 activity in the inflorescence stem is also important for fecundity, most likely by regulating N mobilization and assimilation for effective seed production (Taleski et al., 2020).

In addition, CEPs are important regulators of symbiosis in legumes via the CEPR1 ortholog COMPACT ROOT ARCHITECTURE 2 (CRA2) (Taleski, Jin, et al., 2023). Interestingly, Lotus SGN pathway mutants show reduced expression of *CEP1* and CEP1 application can partially rescue their defective nodule phenotype, suggesting a tight integration of CEP and CIF signaling (Shen, Micic, et al., 2025). CEPs also intersect with CLE signaling for the regulation of symbiosis. CEP‐CRA2 promotes, while CLE signaling inhibits accumulation of the microRNA miR2111, a positive regulator of nodulation (Gautrat et al., 2020; Tsikou et al., 2018). Moreover, CEP expression is sensitive to low phosphate and the CEP‐CRA2 module promotes AMF symbiosis in Medicago (Pedinotti et al., 2024).

In addition, CEPs can promote immune responses against the bacterial pathogen *Pto*. CEPs induce hallmark PTI outputs, including calcium influx, MAPK phosphorylation, and expression of the defense marker gene *FLAGELLIN‐INDUCED RECEPTOR KINASE 1* (Rzemieniewski et al., 2024). Consistently, higher order *CEP* mutants are more susceptible to infection with *Pto*. Surprisingly, immuno‐modulation required CEP activity in the shoot, despite their low expression in above‐ground tissue (Rzemieniewski et al., 2024). CEPs are categorized into two sequence‐divergent groups: group I and group II CEPs with 12 and 3 members, respectively, which are highly conserved at the peptide C‐terminus but show more sequence divergence at the N‐terminus (Delay et al., 2013; Rzemieniewski et al., 2025). Group I contains two non‐canonical members, CEP4 and CEP12, containing two instead of three proline residues and an unusual positioning of the second proline. CEP4 was found to be the most potent inducer of immune outputs on a whole tissue level. Loss of *cepr1* and *cepr2* affected CEP4 sensitivity, and mutation of both was required to confer enhanced susceptibility to *Pto* infection, suggesting an important role for CEPR2 in immunity‐related CEP sensing. CEP4 perception appears unique among CEPs since full sensitivity required the CEPR1/CEPR2‐related RECEPTOR‐LIKE KINASE 7 (RLK7). Canonical members induced immune outputs but do not require RLK7, suggesting distinct ligand specificities among CEP‐CEPR1/CEPR2/RLK7 tissue‐specific modules. Interestingly, perception of canonical CEPs by CEPR1/CEPR2 promoted flg22‐triggered PTI and *Pto* resistance in Arabidopsis seedlings grown under lower external N supply, suggesting that CEPs can integrate immunity with the plant's nutritional status (Rzemieniewski et al., 2024).

In contrast to group I CEPs, the role of group II CEPs remained largely unknown. The group II member CEP14 recently emerged as a novel phytocytokine in Arabidopsis. Its expression is enhanced after MAMP treatment and *Pto* infection. CEP14 promotes immune responses by inducing MAPK phosphorylation, calcium influx and expression of immune marker genes (Rzemieniewski et al., 2025; Wang, Yu, et al., 2024). Moreover, CEP14 is important for systemic acquired resistance (SAR), similar to group I CEPs (Rzemieniewski et al., 2024; Wang, Yu, et al., 2024). CEP13 and CEP15 also showed such activities and mutation of all three group II CEPs conferred enhanced susceptibility to *Pto* (Rzemieniewski et al., 2025). CEP14 interacts with CEPR2 and induces CEPR2‐BAK1 complexes for signal initiation (Wang, Yu, et al., 2024). CEP13 and CEP15 also depend on CEPR2 (Rzemieniewski et al., 2025), revealing additional layers of complexity for diverse sequence‐specific CEP‐CEPR modules. CEPR2 has additional roles in abiotic stress by acting as a negative regulator of ABA responses (Zhang et al., 2021). Whether this role is linked to CEP perception, however, requires further investigation. In wheat, *Ta*CEP15 expression is controlled by the TaZIP9 transcription factor and repressed by drought. *Ta*CEP15 modulates root architecture via the CEPR2 orthologue *Ta*CEPRL to reduce TaSNRK1α stability, a positive regulator of drought stress (Yang et al., 2025). Yet, the wheat pathway appears to play no role for ABA‐induced stomatal closure, suggesting distinct roles in Arabidopsis and wheat.

Collectively, CEP emerge as critical regulators of growth, symbiosis and multiple stress responses. Since CEPs are widely conserved among seed plants, including all major crops, understanding their multifunctional wiring may help future crop improvement strategies (Furumizu et al., 2023).

## PEPs AS PHYTOCYTOKINES WITH EMERGING FUNCTIONS IN ABIOTIC STRESS AND TISSUE REGENERATION

PLANT ELICITOR PEPTIDEs (PEPs) are transcriptionally upregulated during infection or upon elicitor treatment to modulate immunity (Bartels & Boller, 2015). PEPs are produced as propeptides without a signal peptide and their extracellular perception requires non‐canonical secretion. PROPEP1 localizes at the vacuolar vicinity. Upon damage, calcium influx, likely from external and internal stores, activates METACASPASE 4 (MC4) to induce PEP1 maturation. MC4‐dependent PEP release depends on the SUMO E3 ligase SAP AND MIZ1 DOMAIN‐CONTAINING LIGASE 1 (SIZ1)‐mediated PROPEP SUMOylation (Zhang, Wu, et al., 2025). Mature PEP1 may then diffuse to the cell exterior to fulfill its signaling function. In Arabidopsis, PEPs are perceived by two receptors, PEP RECEPTOR 1 (PEPR1) and PEPR2 (Bartels & Boller, 2015). PEP perception is pH‐sensitive. Low pH‐dependent protonation of Asp and Glu residues in the PEPR1‐PEP binding interface disrupts ligand interaction (Liu, Song, et al., 2022). By contrast, RGF1‐RGI‐dependent meristem cell number increase is promoted at acidic conditions with the RGF1 sulfotyrosine residue serving as a pH sensor (Liu, Song, et al., 2022). Extracellular alkalinization is a critical physiological output of immune activation, suggesting that this response further promotes PEPR1‐PEP‐dependent immunity and inhibits RGF‐RGI‐dependent growth in a phytocytokine crosstalk between stress responses and growth regulation.

PEP signaling promotes defense responses upon pathogen‐dependent depletion of the common LRR‐RK co‐receptor BAK1 (Yu et al., 2023). In the absence of BAK1, the LRR‐RK BACK TO LIFE 2 (BTL2) acts as a co‐receptor for both PEP‐PEPR1 and SCOOP‐MIK2 signaling (Yu et al., 2023). BAK1 phosphorylates BTL2 to inhibit its function. Absence of BAK1 alleviates BTL2 inhibition, which then forms complexes with phytocytokine receptors to induce strong immunity culminating in NLR‐dependent cell death (Yu et al., 2023). The structural basis of BTL2 as a PEPR/MIK2 co‐receptor remains, however, unknown. BTL2 has a large extracellular domain with 20 leucine‐rich repeats, raising the question of whether the protein itself may perceive a yet undiscovered endogenous ligand to modulate its activity.

PEP signaling is also associated with growth regulation by for example, controlling auxin fluxes in roots resulting in root growth inhibition and premature root hair formation (Jing et al., 2019). Root growth inhibition is also induced upon flg22 and other MAMP treatments, but PEP1‐triggered root growth responses appear mechanistically distinct (Jing et al., 2019). It remains however unknown whether this is merely explained by the growth–defense trade‐off or may fulfill important physiological functions in the absence of stress. PEP1‐induced changes in root physiology and anatomy are dependent on a transcription factor network involving SALT TOLERANCE ZINC FINGER (STZ) (Dhar et al., 2025). Mutation in *STZ* and related transcription factors alleviated PEP1‐induced root growth inhibition but it remains unknown to which extent this affects PEP1‐induced immunity (Dhar et al., 2025). Fine‐tuning STZ expression levels may be useful to engineer plants for stress adaptation with limited impact on growth. Interestingly, growth‐related PEP functions may also be attributed to another receptor pathway. SUCROSE‐INDUCED RECEPTOR KINASE 1 (SIRK1) directly binds PEP7 to form a co‐receptor complex with QIAN SHOU KINASE 1 (QSK1) to regulate aquaporin phosphorylation, cell swelling and lateral root growth (Wang et al., 2022). QSK1 has various functions in other physiological contexts and stress, including nitrate sensing, immune signaling and camalexin secretion during defense (Aryal et al., 2023; Goto et al., 2024; Zhu et al., 2024). PEP7‐SIRK1 pathway appears to play no role in QSK1‐dependent negative regulation of PTI (Goto et al., 2024) but it remains unknown whether PEP7‐SIRK1 may affect other QSK1‐regulated pathways (Rzemieniewski & Stegmann, 2023).

Furthermore, PEP signaling is involved in sensing the cell wall status. Some PEPs are transcriptionally induced upon cell wall perturbation which is alleviated by MC4‐SIZ1‐dependent PEP maturation (Engelsdorf et al., 2018; Zhang, Wu, et al., 2025). Importantly, recent evidence sheds a new light on PEP roles in plant physiology. The tomato PEP homolog REGENERATION FACTOR 1 (REF1) is an important signal facilitating regeneration upon wounding (Yang et al., 2024). Tissue damage induces *REF1* expression, which then binds to the tomato PEPR1/2 ORTHOLOG RECEPTOR‐LIKE KINASE 1 (PORK1). REF1‐PORK1 induces a pathway activating the transcription factor WOUND‐INDUCED DEDIFFERENTIATION 1 (WIND1) to promote regeneration (Yang et al., 2024). REF1 application triggered callus formation upon stem cuttings in a PORK and WIND1‐dependent manner (Yang et al., 2024). REF‐dependent tissue regeneration appears to be conserved across different plant species and may be applied to promote tissue regeneration in recalcitrant crops as a bottleneck for genetic engineering (Yang et al., 2024). The role of PEPs in immunity and tissue repair suggests a dual function in defense activation and re‐establishment of tissue integrity once danger from intruders has ceased. Collectively, this suggests that PEPs operate at the interface between stress responses, growth and regeneration as central players for plant organismal homeostasis. How the multifunctional roles of PEPs are mechanistically connected remains an important future research question.

## RALFs AS CENTRAL REGULATORS OF PHYSIOLOGY WITH NOVEL STRUCTURAL FUNCTIONS

**Figure 2:**
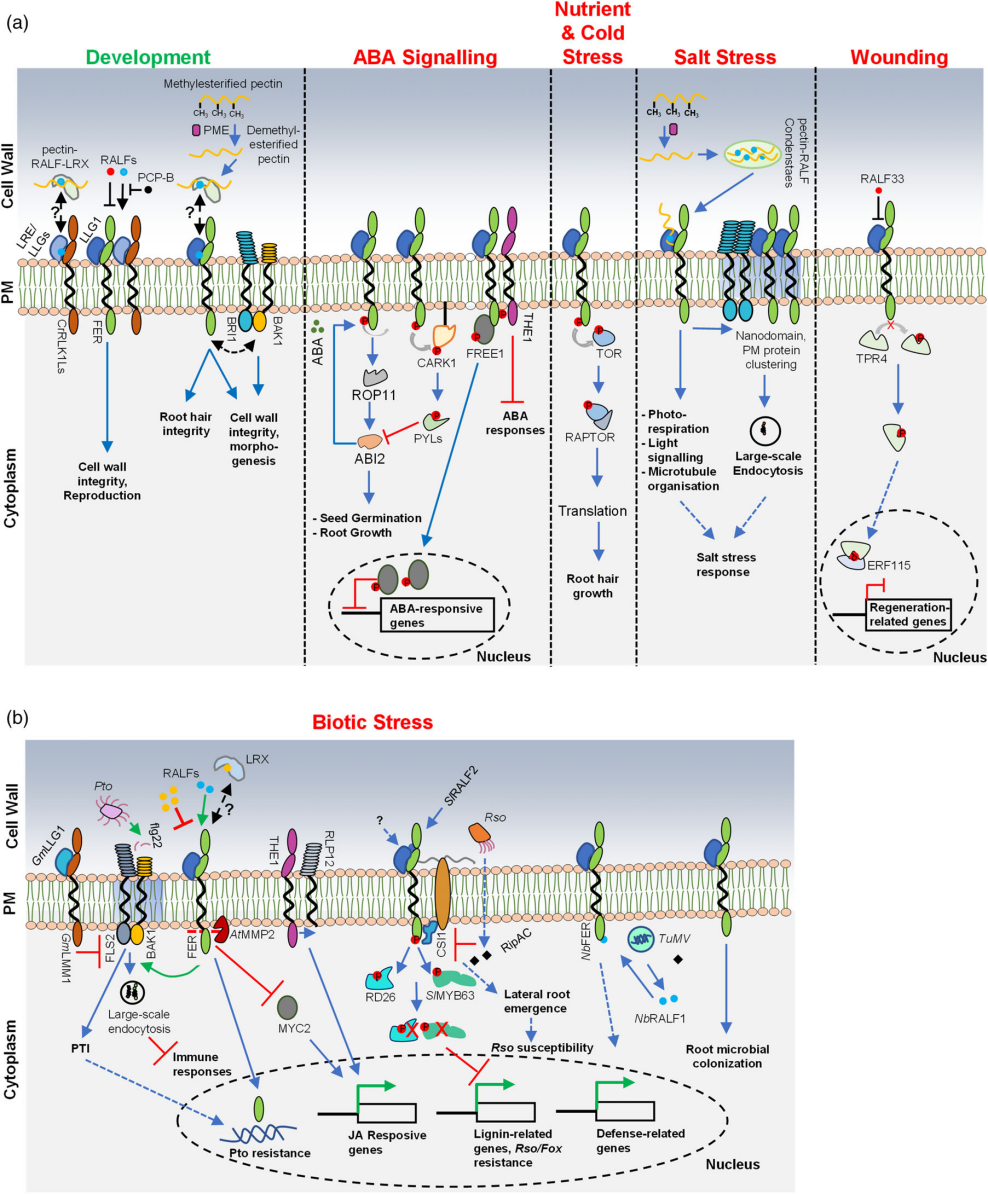
Role of RALF and RALF‐binding modules in development, abiotic, and biotic stress responses. (a) Development: RALF peptides bind to demethylated pectin via LRX proteins and engage with CrRLK1L complexes to regulate reproduction, root hair integrity, and cell morphogenesis. Abiotic stress: FER and other CrRLK1Ls modulate ABA signaling (both positively and negatively) through several downstream pathways. Under nutrient limitation and cold stress, FER activates the TOR kinase pathway to control translation. During salt stress, RALF–pectin condensates regulate surface clustering/nanodomain organization of plasma membrane proteins, including BRI1, as well as photorespiration, light signaling and microtubule organization. Upon wounding, FER inhibits regeneration via TPR4 phosphorylation, thereby inhibiting regeneration‐related genes. This FER function is inhibited by RALF33 (b) Biotic stress: FER regulates nanodomain organization of flg22‐induced complex formation of FLS2–BAK1. Furthermore, cleavage of its kinase domain modulates root immune signaling. The THE1–RLP12 complex regulates JA‐responsive genes, while FER‐mediated inhibition of MYC2 fine‐tunes JA responses. In tomato and Arabidopsis roots, RALF‐FER signaling induces degradation of *Sl*MYB63 and RD26, respectively, leading to enhanced lignin biosynthesis and *Rso* or *Fox* resistance, respectively. In addition, *Rso* targets the FER interactor CSI1 to enhance susceptibility via promotion of lateral root emergence. In *Nicotiana benthamiana*, NbRALF1 enhances resistance against TuMV by binding to the intracellular kinase domain of NbFER. FER also regulates the rhizosphere microbiome composition. Solid arrows denote positive regulation, headless arrows negative regulation.

RAPID ALKALINIZATION FACTORs (RALFs) are cysteine‐rich peptides and central regulators of multiple aspects of plant physiology, including development and reproduction (Figure 2a) (Cheung, 2024). RALFs bind to plasma membrane heterocomplexes of LORELEI/LORELEI‐LIKE GPI‐ANCHORED PROTEINs (LRE/LLGs) and CATHARANTHUS ROSEUS RECEPTOR‐LIKE KINASE 1‐LIKEs (CrRLK1Ls) to induce downstream responses (reviewed in Cheung (2024)). CrRLK1Ls frequently form multi‐member complexes but their spatiotemporal organization remains largely unclear (Schoenaers & Vissenberg, 2025). The best described and most studied CrRLK1L is FERONIA (FER), which is involved in a multitude of physiological outputs (Cheung, 2024). A key function of CrRLK1L‐RALF‐LLG complexes is to monitor cell wall integrity and in that framework, they regulate a diverse array of growth responses. For example, in vegetative tissues, RALF‐FER regulates hypocotyl elongation and cell morphogenesis in an interplay with brassinosteroid signaling (Biermann et al., 2025; Chaudhary et al., 2025; Lu et al., 2024). For successful fertilization, CrRLK1L‐RALF‐LLG modules control pollen hydration, tube elongation, polytubey blockages and pollen tube rupture upon arrival at the synergid cells (Duan et al., 2020; Ge et al., 2017; Lan et al., 2023; Liu et al., 2021; Liu et al., 2025; Zhong et al., 2022). During reproduction, CrRLK1L complexes are regulated by antagonistic RALFs and further modulated by RALF‐unrelated POLLEN COAT B‐TYPE PROTEINS (PCP‐Bs), suggesting that these complexes can perceive multiple ligands to fine‐tune response outputs (Lan et al., 2023; Liu et al., 2021; Liu et al., 2025).

In addition, CrRLK1L pathways are central for various biotic and abiotic stress responses. FER functions as a context‐specific positive or negative regulator of ABA signaling (Figure 2a). FER promotes activation of the PP2C phosphatase ABA INSENSITIVE 2 (ABI2) to inhibit ABA responses and enhance seed germination and root growth. ABI2 in turn dephosphorylates FER, which is relieved upon RALF1 recognition (Chen et al., 2016). FER phosphorylates FYVE DOMAIN PROTEIN REQUIRED FOR ENDOSOMAL SORTING 1 to induce nuclear translocation and suppression of ABA response gene expression (Fu et al., 2025). Surprisingly, during early seed germination and radicle protrusion, FER promotes ABA signaling by phosphorylating the RLCK CYTOSOLIC ABA RECEPTOR KINASE 1 (CARK1), which phosphorylates PYRABACTIN RESISTANCE1 (PYR1)/PYR1‐LIKEs (PYLs)/REGULATORY COMPONENTS OF ABA RECEPTOR (RCARs) to promote ABI2 inhibition (Wang, Liu, et al., 2024). A link of RALF perception to CARK1 phosphorylation remains, however, unknown. THESEUS 1 (THE1) coordinates cell wall integrity with hormonal responses by promoting ISX‐induced JA signaling via RLP12 and inhibition of turgor‐dependent ABA responses (Bacete et al., 2021). This highlights the tight correlation between CrRLK1L modules and ABA‐dependent abiotic stress. During nitrogen starvation and cold stress, FER regulates protein translation by regulating activity of the central growth regulator TARGET OF RAPAMYCIN (TOR) (Pacheco et al., 2023; Song et al., 2022). FER further regulates salt tolerance by coordinating photorespiration, PHYTOCHROME B‐dependent red light signaling, microtubule organization, and global plasma membrane RK and proton pump endocytosis. FER and additional CrRLK1Ls further regulate fertility under heat stress in cotton via RALF‐mediated surface receptor clustering (Jiang et al., 2024; Liu et al., 2023; Liu, Wang, et al., 2024; Liu, Yeh, et al., 2024; Zhang, Li, et al., 2025). Recent evidence connects FER‐RALF modules to tissue regeneration upon physical damage. FER inhibits root regeneration upon cutting by phosphorylating the transcriptional co‐repressor TOPLESS‐RELATED 4 (TPR4) that sequesters ETHYLENE RESPONSE FACTOR 115 (ERF115) in the cytoplasm. Upon tissue damage, RALF33 is secreted, which inhibits FER‐mediated TPR4 phosphorylation, resulting in ERF115 nuclear accumulation and the induction of regeneration‐related genes (Shen, Xie, et al., 2025).

CrRLK1L modules are also central regulators of immunity and plant–microbe interactions (Figure 2b). FER, together with LLG1, functions as a RALF‐regulated scaffold controlling PRR nanoscale trajectories within the plasma membrane for the assembly of PRR complexes (Gronnier et al., 2022; Stegmann et al., 2017; Xiao et al., 2019). RALF23 inhibits FLS2‐BAK1 receptor complex formation and affects nanoscale organization of FLS2 and BAK1 in the plasma membrane via FER‐LLG1 (Gronnier et al., 2022; Stegmann et al., 2017). RALF23 suppresses FER resulting in stabilization of the JA transcription factor MYC2 to inhibit *Pto* resistance (Guo et al., 2018). In roots, RALF23 perception by FER promotes metalloprotease AtMMP2‐dependent cleavage of the FER kinase domain which translocates to the nucleus to regulate resistance against *Pto* (Chen et al., 2024). The RALF23‐related RALF1 induces FLS2‐FER co‐clustering at the PM and subsequent endocytosis which may contribute to RALF‐mediated immune modulation (Liu, Yeh, et al., 2024). RALF23 effects on FLS2 signaling appear to be concentration‐dependent, since low doses of RALF23 promote flg22‐induced FLS2‐BAK1 interaction, while high levels inhibit this function (Chen et al., 2024). It will be important to reveal whether dosage‐dependent differential RALF effects extend to other CrRLK1L‐regulated physiological outputs. Also, some RALFs were reported to promote immunity via FER, raising the question whether antagonistic RALF regulation extends to multiple FER/CrRLK1L‐mediated pathways beyond reproduction (Abarca et al., 2021; Lan et al., 2023; Stegmann et al., 2017; Zhong et al., 2022). For example, RALF22 perception by FER promotes PEP3‐triggered immunity against *Sclerotinia sclerotiorum* in Arabidopsis (He et al., 2023). The FER‐RALF module for the control of root immunity is influenced by nutrient conditions and coordinates beneficial and commensal root microbial colonization (Song et al., 2021; Tang et al., 2022). Other CrRLK1Ls also regulate several layers of the plant immune system but a connection to specific RALF ligands remains unknown (Ortiz‐Morea et al., 2022).

Surprisingly, FER's role in immunity appears to be species, tissue and/or microbe‐specific. For example, FER is a negative regulator of disease resistance against the root and vasculature infecting pathogen *Ralstonia solanacearum (Rso)*. FER phosphorylates and destabilizes the transcription factor RESPONSE TO DESICCATION 26 to inhibit lignin accumulation in the vasculature in Arabidopsis (Wang, Luo, et al., 2024). Also, *Rso* secretes the RipAC effector protein that disrupts cellulose biosynthesis to promote lateral root emergence as pathogen entry sites by interacting with CELLULOSE SYNTHASE INTERACTIVE PROTEIN 1 (CSI1) and FER (Yu et al., 2024). Similarly, tomato FER promotes degradation of the MYB63 transcription factor in response to RALF2 perception to inhibit lignin accumulation and resistance against *Fox* (Yanfen et al., 2024). Arabidopsis FER mutants are more resistant to powdery mildew infection and the function of multiple endogenous RALFs is required for powdery mildew's asexual life style completion (Kessler et al., 2010; Leicher et al., 2025). Surprisingly, the soybean FER and LLG1 homologs GmLMM1 and GmLLG1/GmLLG2 function as negative regulators of PTI and resistance against *Pto* infection (Wang, Chen, Wang, et al., 2025). This highlights the need to study FER pathways in several species before potential applications for crop improvement. FER also regulates resistance against viral infections. In *Nicotiana benthamiana*, NbRALF1 promotes *Nb*FER‐dependent resistance against turnip mosaic virus (TuMV). Interestingly, defense induction against TuMV requires RALF1 perception by the intracellular kinase domain of *Nb*FER (Rui et al., 2025). It will be important to reveal whether intracellular recognition of RALFs by FER and other CrRLK1Ls is more widespread and how this is coordinated with canonical extracellular perception.

Importantly, our understanding of the biochemical and physiological function of RALF peptides was recently put into new light. LEUCINE‐RICH REPEAT EXTENSIN proteins are high affinity RALF‐binding proteins that are involved in FER‐RALF‐regulated physiological outputs (Schoenaers & Vissenberg, 2025). In Arabidopsis, LRX8 forms homodimers and binds RALF4 with high affinity, particularly at acidic conditions via RALF4's C‐terminus. RALF4 is closely related to RALF23 which binds to LLG proteins via an N‐terminal YISY motif (Moussu et al., 2020; Xiao et al., 2019). Importantly, RALF4 binding to LRX8 exposes a polycatinoic tail on RALF4 that allows interaction with demethylated, negatively charged pectin moieties in the cell wall. Similarly, the related RALF1 forms pectin condensates (Liu, Yeh, et al., 2024). LRX8‐RALF4 and LRX1‐RALF22 complexes induces pectin compaction and form reticulated networks surrounding growing pollen tubes and root hairs, respectively, suggesting a function as integral structural cell wall components (Moussu et al., 2023; Schoenaers et al., 2024). Interestingly, *RALF22* root hair expression is induced by ethylene released from the fungal pathogen *Penicillium aurantiogriseum*, suggesting its capacity to coordinate root growth and integrity with the biotic environment (Morcillo et al., 2024). Importantly, FER‐dependent RALF signaling outputs require association between RALFs and demethylated pectin, suggesting a tight interconnection between RALF's cell wall structural and signaling functions. The pectin methylation status and thus pectin's ability to interact with RALFs is determined by PECTIN METHYLESTERASE (PMEs). PME activity is in turn modulated by PME INHIBITORS (PMEIs) in a pH‐dependent manner (Xu et al., 2022). RALF1‐induced plasma membrane receptor clustering and cell invaginations, RALF1 and RALF23‐induced root growth inhibition, as well as RALF23 promoted brassinosteroid signaling are compromised by PMEI overexpression, chemical interference with PME activity or by promotion of pectic fragment degradation (Biermann et al., 2025; Liu, Yeh, et al., 2024; Rößling et al., 2024). The mechanistic basis for the interplay between RALFs structural and signaling function, however, remains little understood and is an important future research question (Schade et al., 2025; Schoenaers & Vissenberg, 2025). A hallmark output of CrRLK1L‐RALF signaling is apoplastic alkalinization via H + ATPase phosphorylation and endocytosis, as well as proposed proton influx regulation via yet unknown channels (Haruta et al., 2014; Li et al., 2022; Liu, Yeh, et al., 2024). This raises the question whether CrRLK1L‐dependent pH modulation is part of a rheostat mechanism to safeguard appropriate RALF cell wall integration.

## MULTIPLE OTHER PEPTIDE RECEPTOR MODULES OPERATE AT THE INTERSECTION OF GROWTH AND STRESS RESPONSES

Similar to PEPs, PAMP‐INDUCED PEPTIDEs (PIPs) are transcriptionally induced upon infection and elicitor treatment (Hou et al., 2014). They promote immunity and are important for resistance against pathogens in Arabidopsis and other plant species (Hou et al., 2014). PIPs are perceived by RLK7 in a BAK1‐dependent manner to induce immunity (Hou et al., 2014). PIPs are sequence‐related to CEPs and RLK7 binds to both CEP4 and PIP1, although PIP1 binding shows stronger affinity (Rzemieniewski et al., 2024). Thus, it remains important to reveal the physiological interplay of differential RLK7 ligands. PIP3‐RLK7 promotes salt tolerance in Arabidopsis (Zhou et al., 2021). PIP‐LIKE 3 (PIPL3)/TARGET OF LBD SIXTEEN 2 (TOLS2) is transcriptionally induced by auxin at lateral root primordia and reduces LR initiation and spacing. TOLS2 is perceived by RLK7 and regulates the activity of the transcription factor PUCHI (Toyokura et al., 2019). PIP2 also induces RLK7‐dependent immune outputs and its overexpression promotes *Fox* resistance (Hou et al., 2014). The specificity of distinct PIP modules for immune and growth regulation requires further investigation. Interestingly, TOLS2 signaling relies on CIK1/2/4/5/6 co‐receptors (Meng et al., 2024). This suggests that RLK7 can recruit ligand‐specific co‐receptors, which may allow the specification of distinct downstream outputs.

INFLORESCENCE DEFICIENT IN ABSCISSION (IDA) and IDA‐LIKE (IDL) are regulators of organ abscission via HAESA (HAE) and HAE‐LIKEs (HSLs) (Li & Su, 2024). HAE‐IDA signaling is also associated with the regulation of immune responses (Lalun et al., 2024). Interestingly, HSL3 perceives very different endogenous peptides to regulate stress responses. HSL3 is phylogenetically related to HAE/HSLs but lacks key conserved motives required for IDA/IDL binding (Rhodes et al., 2022). HSL3 perceives CTNIP/SMALL PHYTOCYTOKINES REGULATING DEFENSE AND WATER LOSS (SCREW) peptides to regulate stomatal immunity and was thus renamed PLANT SCREW‐RESPONSIVE RECEPTOR (NUT). CTNIP/SCREWs are sequence unrelated to IDA/IDLs/CEPs/PIPs and contain two conserved cysteine residues that are required for receptor binding. Upon CTNIP/SCREW perception, a HSL3/NUT‐BAK1 complex induces immune signaling outputs, including ROS production, MAPK phosphorylation and *Pto* resistance. Immune activation upon flg22 perception triggers stomatal closure, but this can be problematic since this generates aqueous environments for bacterial proliferation upon entry. SCREW‐NUT signaling facilitates stomatal reopening after invasion by counteracting stomatal closure (Liu, Hou, et al., 2022). The *nut* mutants also display increased ABA sensitivity and tolerance to drought, suggesting that this pathway can integrate abiotic and biotic stress to optimize plant performance under adverse environmental conditions (Liu, Hou, et al., 2022). However, a role for development remains unknown.

## FUTURE QUESTIONS

This review highlights that multiple peptide pathways operate at the intersection of growth responses, nutrient sensing, and stress mitigation, raising important questions about their mechanistic interconnection (Box 2).

Box 2 **Open questions**

- How do multifunctional receptor‐peptide modules achieve signaling specificity to define distinct physiological outputs?
- How does the spatiotemporal availability of agonist/antagonist ligand cocktails define output specificity?
- How do distinct receptor modules that regulate similar physiological outputs converge mechanistically?
- How can we harness knowledge on peptide modules at the intersection of growth, nutrient sensing, and stress responses for crop improvement strategies?

Several physiological pathways are regulated by multiple peptide signaling modules. How do these pathways intersect in space and time? Several receptors perceive antagonistic ligands. For example, the tomato phytocytokine SYSTEMIN is regulated by an endogenous antagonist (Wang, Maier, et al., 2025). How do individual receptors determine appropriate outputs based on differential ligands? How is output specificity achieved through receptors with overlapping expression patterns and antagonistic functions?

Most, if not all, RK pathways rely on similar receptor complex components and downstream executors. How can individual receptor complexes determine downstream specificity despite their similar reliance on conserved signaling executors? Part to the answer may lie in differential combinations of specific executors. An example is the identification of distinct MAPK pathways operating downstream of FLS2 and SGN3 for specifying casparian strip closure vs. immunity on a single‐cell level in the root endodermis (Ma et al., 2024). This in turn may be determined by RLCKs that operate upstream of MAPKs. Subfamily VII in Arabidopsis has a particularly prominent role operating downstream of multiple receptors. Here, individual members can define specificity toward immunity versus growth (DeFalco et al., 2022).

Emerging evidence suggests that dephosphorylation can kick‐start RK pathways, including activation of SYSTEMIN RECEPTOR 1 and PSKR1 during aluminum ion sensing (Li et al., 2025; Xu et al., 2025). What is the exact timing of events to initiate downstream outputs in an interplay between kinases and phosphatases and may this translate into outcome specificity? Peptide‐perceiving RKs display distinct nanodomain organization and trajectories within individual plant cells (Gronnier et al., 2022). How does this translate to multiple receptors present in the plant cell at the same time and downstream (shared) executors? Are the multitude of complex components identified for individual RKs always present or do complexes show distinct spatiotemporal organizations?

General hubs emerge that can integrate growth and stress responses, including CPK28 that determines growth versus immunity downstream of PSKR1 (Ding, Lv, et al., 2022). How can we harness such knowledge for crop improvement strategies? Can we modulate hub proteins to promote resistance without compromising growth through selective engineering?

To address these questions a combination of genetic, biochemical, and advanced imaging technologies will be necessary. Also, it will be important to harness knowledge of modern omics approaches, including single‐cell RNA sequencing, technologies that show emerging insights into plant stress responses (Nobori et al., 2024).

## Conflict of Interest

The authors declare no conflict of interest.

## Data Availability

Data sharing not applicable to this article as no datasets were generated or analysed during the current study.
